# Supplementary material for: Chromothripsis during telomere crisis is independent of NHEJ, and consistent with a replicative origin
Source: Genome Res. 2019 May;29(5):737–49. doi: 10.1101/gr.240705.118 (PMC6499312; doi:10.1101/gr.240705.118)
Supplement: Supplemental Material [file supp_gr.240705.118_Supplemental_file_1.zip › contigs/annotated_contigs/DB51/contig.2.DB51_length_726_mean_cov_4.53994490358.docx]

**DB51_length_726_mean_cov_4.53994490358**

```
TTTGCAAAGGCAGTTTCAATATGGCACTAGAGAAATCTGCAAGCAAACCGTATTCCATCAGTTCGCTGTCATATATTTCCCTTCTCACA
 >chr18:68691653-68691955 - E=7e-170 p=5e-03
GTTTGCCACTCTTAGAAGCCTAAATTCACTTTTCCTTGTCCTGTCATTTTCTACAAATGTATTGTCTTTTGTTAAAGATGCTGTATAAG

TCAGAGCCCTAAGCCACCTCTTCAAGTTACTAATTTTCTTGGGTGTTTCCCATGTACATATGAGATAAATATGTTAATAAACTTGTTTG

TTTTTCTGTAGCTAATCTGTCTTTTATTAGAG|GGA|GTAGTATCACATGTGCTTATTGGTCATTTATAGAAATTCTTGTTTTTTTTGA
                                 >chr18:68705862-68706264 + E=3e-207
ATTGCTAACATCTTTTGCCCACTAAAAAAAATTGAAATGTCTTTTTATTTTCGATTTAAGCCAAGTTTTTAATATTTGTTACTATATAC

AAATCCGTTTTCAGATATATTTATTGCAAATATTTTCTGCCCATCTGCTGGTTTTCCTTGTCACTTTCTCAACAGAGTTTGGGCAAACA

AAAGTTTTTAATTTTGTTGAAGTTCAGTTTATCCATGTTTTCTTAAATGGTCAGTGCTTTTTGTGTCCTATCTTAAATATTTTCTTAAA

CTTAAAATATTTTCTTCTACATTTTCTTCTAGGTGATTTGGAGTTTTCTTTTTGTTGTTTATGCATACATTGTGACTCA|GATTTATGC

TTTTCTGTTGTGAGATC
```
